# Therapeutic Issues Associated with Gender-Affirming Hormone Therapy in Transgender Individuals: A Systematic Review Relevant to Community Pharmacy Practice

**DOI:** 10.3390/pharmacy14060132

**Published:** 2026-09-10

**Authors:** Fabrice Mitoumba, Héloïse Henry, François Medjkane, Sophie Gautier, Bertrand Décaudin

**Affiliations:** 1 ULR 7365-GRITA-Groupe de Recherche sur les Formes Injectables et les Technologies Associées, Université de Lille, F-59000 Lille, Franceheloise.henry@univ-lille.fr (H.H.); 2 CHU Lille, Institut de Pharmacie, F-59000 Lille, France; 3 CHU Lille, Dispositif Transidentité(s), Service de Psychiatrie de L’enfant et de L’adolescent, Hôpital Fontan, Faculté de Médecine, Université de Lille, F-59000 Lille, France; 4 CHU Lille, Service de Pharmacologie Médicale, Centre Régional de Pharmaco-Vigilance du Nord Pas de Calais, Faculté de Médecine, Université de Lille, F-59000 Lille, France; sophie.gautier@chu-lille.fr

**Keywords:** community pharmacy, gender-affirming healthcare, medication safety, pharmaceutical care, systematic review, transgender persons

## Abstract

Community pharmacists play an increasingly important role in the care of transgender individuals receiving gender-affirming hormone therapy (GAHT). However, the content of structured pharmaceutical consultations tailored to this population has not yet been clearly defined. This systematic review aimed to identify therapeutic issues relevant to community pharmacy practice in order to inform the development of structured pharmaceutical consultations. A systematic literature review was conducted in accordance with the PRISMA 2020 Statement. The review question was structured using the PICo framework, with transgender individuals as the population, treatment-related issues associated with GAHT as the phenomenon of interest, and community pharmacy practice as the context. Electronic searches of Medline, Google Scholar, and APA PsycINFO were performed on 17 July 2024. Studies involving transgender adolescents or adults receiving GAHT and reporting therapeutic effects, adverse events, monitoring requirements, or medication-related issues were eligible. Two reviewers independently screened the studies and extracted data using a standardized form. Owing to the heterogeneity of study designs, the findings were synthesized narratively according to therapeutic domains relevant to community pharmacy practice. Among 755 screened records, 116 articles met the inclusion criteria. Seven therapeutic domains relevant to pharmacist-led consultations were identified: cardiovascular and cardiometabolic effects, dermatologic effects, gynecologic effects, musculoskeletal effects, biological and pharmacological effects, psychiatric effects, and other adverse effects or specific clinical situations. The evidence highlighted treatment-specific monitoring needs, medication-related risks, fertility and contraceptive considerations, laboratory monitoring requirements, drug–drug interactions, and mental health outcomes. Community pharmacists may contribute to the monitoring and optimization of GAHT, adverse effect management, patient education, fertility and contraceptive counseling, pharmacovigilance, and interdisciplinary collaboration within the scope of their professional practice. These findings provide an evidence-informed basis for the future development of structured pharmaceutical consultations tailored to transgender individuals.

## 1. Introduction

Transgender healthcare engages all primary healthcare professionals, including community pharmacists [1,2]. Providing safe, effective, and gender-affirming pharmaceutical care presents several clinical and organizational challenges for community pharmacists. The primary objective is to ensure that transgender individuals can access a healthcare pathway that aligns with their personal goals for well-being and self-actualization, while safeguarding both physical and mental health [1]. Community pharmacists play a pivotal role in the initiation and ongoing management of gender-affirming hormone therapy (GAHT), as well as associated pharmacological treatments [3,4]. Numerous clinical considerations are involved in this process. These include ensuring secure and evidence-based access to pharmacotherapy, particularly in response to the risks associated with unsupervised medication use, such as self-prescription, online procurement, or sharing of prescriptions. Pharmacists are also essential in supporting the use of off-label medications when access to specialized care is limited. Their responsibilities extend to patient education, promoting autonomous and informed management of therapy, and addressing clinical queries in a patient-centered manner, with consideration for individual health literacy and resources. Furthermore, community pharmacists must be vigilant regarding the emergence of adverse drug reactions and are instrumental in their identification and management in coordination with the patient and the primary care provider [5,6]. Pharmacovigilance must also encompass potential drug–drug interactions, particularly in cases of polypharmacy.

Given these responsibilities, it is imperative that community pharmacists receive adequate training, consistent with the evolving needs of transgender healthcare [7].

In this context, the World Professional Association for Transgender Health (WPATH) released the Standards of Care—Eighth Edition (SOC-8) in 2022, offering comprehensive, evidence-based guidelines to support healthcare professionals [1]. Among the 18 chapters, Chapter 12 (Gender-Affirming Hormone Therapy) and Chapter 18 (Primary Care) are particularly pertinent to the role of community pharmacists. For instance, recommendation 12.9 emphasizes the importance of regular clinical and laboratory monitoring of GAHT. Additionally, recommendations 15.1 to 15.14 underscore the necessity of coordinated, multidisciplinary care, highlighting specific areas of focus for pharmacists, including smoking cessation counseling and management of concurrent antiretroviral therapy.

While the pharmacist’s involvement is most commonly associated with medication dispensing, there is increasing recognition of the need for structured pharmaceutical consultations tailored to the specific needs of transgender individuals at various stages of their care pathway. Within a multidisciplinary approach, community pharmacists may contribute to medication-related care, patient counseling, and treatment monitoring, while facilitating referral to other healthcare professionals when appropriate and according to their scope of practice. Although pharmaceutical consultations are currently implemented across various clinical domains within community pharmacies, a defined framework for such interventions in the context of transgender care remains lacking. Consequently, synthesizing the available evidence is an essential first step toward informing the future development of structured pharmaceutical consultations dedicated to transgender individuals receiving GAHT.

The objective of this systematic review was to identify and synthesize treatment-related issues associated with gender-affirming hormone therapy in transgender individuals that may be relevant to community pharmacy practice and the development of structured pharmacist-led consultations.

## 2. Materials and Methods

### 2.1. Data Sources and Search Strategy

This systematic review was designed to answer a research question structured according to the PICo framework as follows:

- Population (P): Transfeminine and transmasculine individuals receiving GAHT.
- Interest (I): Treatment-related issues associated with GAHT, including therapeutic effects, adverse events, monitoring requirements, medication-related issues, and other clinical considerations.
- Context (Co): Community pharmacy practice, with a particular focus on identifying evidence to inform the future development of structured pharmacist-led consultations.

The review therefore focused on identifying treatment-related issues relevant to the community pharmacy context rather than evaluating predefined clinical outcomes or the effectiveness of pharmacist-led interventions.

This systematic review with narrative synthesis was conducted in accordance with the Preferred Reporting Items for Systematic Reviews and Meta-Analyses (PRISMA 2020) Statement. The electronic search was conducted on 17 July 2024 using Medline, Google Scholar and APA PsycINFO via EBSCOhost. These databases were selected to provide coverage of biomedical, psychological, and broader interdisciplinary literature relevant to GAHT and pharmaceutical care. Prior to manuscript resubmission, a review of recently published literature was performed to identify relevant evidence that could inform the Discussion; these publications were not incorporated into the systematic review results. The search strategy combined three predefined concepts related to (i) transgender people, (ii) therapeutic issues and pharmaceutical care, and (iii) hormone therapy, using both free-text keywords and Medical Subject Headings (MeSH) where applicable. For Google Scholar, multiple title-based searches were conducted using the “allintitle:” operator and limited to publications from 2012 onwards. All retrieved results were screened for eligibility; no formal stopping criterion was prespecified. The complete search strategy for Medline, APA PsycINFO and Google Scholar is provided in Supplementary File S1. All retrieved records were screened. In addition, the reference lists of all included studies were manually screened to identify potentially eligible publications not retrieved through the electronic search.

### 2.2. Study Selection

Bibliographic records were managed using Zotero, and study screening was performed using Rayyan.

Eligibility criteria were defined a priori. Studies were included if they included transgender adolescents or adults receiving gender-affirming hormone therapy and reported clinical outcomes, adverse events, monitoring parameters, or therapeutic considerations relevant to community pharmacy practice. Eligible studies included original research articles reporting treatment-related effects, adverse events, clinical outcomes, or other therapeutic issues associated with gender-affirming hormone therapy in transgender individuals. All study designs were considered eligible a priori, including prospective and retrospective cohort studies, cross-sectional studies, case–control studies, qualitative studies, case series, case reports, and pharmacovigilance studies. The search was restricted to full-text articles published in English or French. No publication-date restriction was applied to MEDLINE or APA PsycINFO, whereas the Google Scholar search was limited to publications from 2012 onwards. There were no restrictions on participants’ age or geographical location.

Studies were excluded if they (i) did not involve transgender individuals receiving GAHT, (ii) did not report treatment-related effects, adverse events, monitoring parameters, medication-related issues, or other clinical considerations associated with GAHT, (iii) were reviews, editorials, letters, conference abstracts or other non-original publications, or (iv) were published in languages other than English or French. Studies were not required to involve pharmacists or to have been conducted in community pharmacy settings. Relevance to community pharmacy practice was determined according to the nature of the treatment-related issue reported and its potential implications for medication monitoring, counselling, medication safety, or referral within the scope of community pharmacy practice.

The study selection process was conducted in four successive stages. First, records identified through the three databases were retrieved and imported into Zotero. Duplicate records were automatically identified and manually verified before the remaining references were transferred to Rayyan. Two investigators independently screened titles and abstracts to identify potentially eligible studies followed by full-text assessment of potentially eligible studies. Disagreements at any stage were resolved through discussion or, when necessary, by consultation with a third reviewer. Full texts were sought through the electronic databases, available institutional access, and other accessible sources. Reports for which the full text could not be accessed were excluded from the full-text assessment.

### 2.3. Data Extraction and Synthesis

Data extraction was independently performed by two investigators using a standardized extraction form. The following information was collected: first author, publication year, country, study design, sample size, participant characteristics, hormone therapy regimen, outcomes assessed, and principal findings. Any disagreements were resolved through discussion with a third reviewer.

Because the objective of this review was to comprehensively identify and characterize therapeutic issues relevant to community pharmacy practice rather than estimate the effects of a specific treatment or intervention, no formal assessment of methodological quality or risk of bias was performed. Given the heterogeneity of study designs, study populations, hormone regimens, and reported outcomes, a meta-analysis was not considered appropriate. A narrative synthesis was therefore conducted to map the available literature and describe the therapeutic issues and clinical considerations relevant to pharmaceutical care. During the analysis of the included articles, the identified treatment-related issues were inductively organized into seven therapeutic domains reflecting their potential relevance to community pharmacy practice and structured pharmacist-led consultations. The categorization was developed through discussion and consensus among the investigators. Data were summarized in tables reporting study characteristics, objectives, population, outcomes assessed, and principal findings.

The review protocol was submitted to the International Prospective Register of Systematic Reviews (PROSPERO) on 30 October 2025 and formally registered on 11 February 2026 (registration number CRD420251167559) after completion of the initial screening and data extraction.

## 3. Results

**Figure 1:**
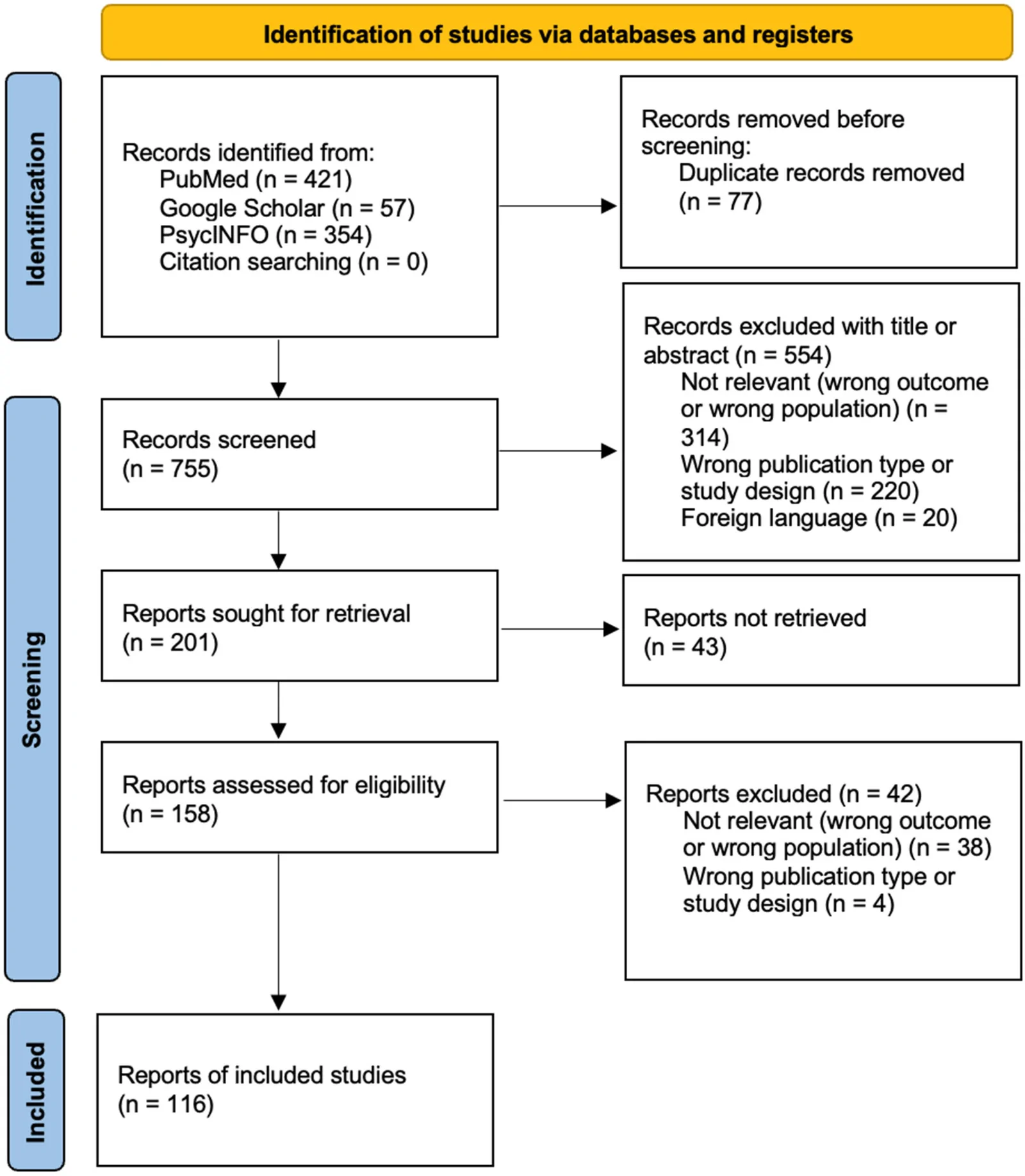
PRISMA flow diagram of the study selection process.

A total of 755 articles were screened after removing duplicates. A total of 116 articles met the inclusion criteria (Figure 1).

The studies were grouped into 7 areas of potential relevance to pharmacist-led consultations: cardiovascular effects and cardiometabolic risks (37 studies), dermatological effects (4 studies), gynecological effects (8 studies), musculoskeletal effects (11 studies), biological and pharmacological effects (32 studies), psychiatric effects (11 studies) and other side effects and specific clinical situation (13 studies) (Supplementary Tables S1–S7). Although these domains were not mutually exclusive, each included study was assigned to a single primary domain for reporting purposes. The included studies primarily addressed treatment-related issues associated with GAHT in transfeminine and transmasculine individuals, while the community pharmacy context provided the framework for interpreting the potential relevance of these findings to pharmacist-led consultations. The main treatment-related issues identified across the seven domains, together with their potential relevance to community pharmacy practice, are summarized in Supplementary Table S8.

### 3.1. Cardiovascular Effects and Cardiometabolic Risks

The reviewed literature demonstrates heterogeneous findings regarding cardiovascular and metabolic risks associated with GAHT (Supplementary Table S1). Large cohort studies reported an increased incidence of venous thromboembolism (VTE) and stroke in transfeminine individuals compared with cisgender controls, while risks in transmasculine cohorts were less consistent [8,9]. Other population-based analyses observed modestly elevated cardiovascular disease rates in both transfeminine and transmasculine individuals, though absolute risks remained relatively low and often involved benign arrhythmias [10]. Retrospective chart reviews in adolescents and young adults showed no acute thrombotic events linked to GAHT [11]. In a pharmacovigilance study of transgender men taking testosterone enanthate, six patients with reported adverse drug reactions (ADRs) were identified; all reported ADRs were cardiovascular or thromboembolic events, which included deep vein thrombosis (n = 1), pulmonary embolism (n = 3; 50%), and ischemic stroke (n = 2) [6]. Lipid profile changes diverged by sex assigned at birth: GAHT improved lipid parameters in transfeminine individuals but produced atherogenic changes in transmasculine individuals [12,13]. Case reports described rare but severe cardiovascular events, including spontaneous coronary artery dissection and retinal vein occlusion [14,15]. Current evidence on endothelial function in transmasculine individuals remains limited [16]. Several studies emphasized increased obesity and BMI, particularly among transmasculine youth [17,18]. Overall, the evidence suggests that GAHT is associated with differential cardiometabolic effects based on treatment type and population subgroup, warranting long-term prospective studies.

### 3.2. Dermatologic Effects

The reported dermatologic effects of GAHT varied across the included studies (Supplementary Table S2). In transmasculine individuals, acne prevalence rose significantly after initiation of masculinizing hormone therapy, affecting nearly one-third of patients after a mean of 3.4 years of treatment, with the highest risk observed among those aged 18–21 years [19]. The same cohort demonstrated an increased incidence of androgenetic alopecia, particularly after the fourth year of therapy [20]. In contrast, feminizing therapy in transfeminine individuals was associated with persistent excess facial and body hair despite treatment, with 84% of transfeminine individuals on hormones and 100% of those not on therapy reporting this concern; nonbinary individuals on feminizing therapy reported a similar prevalence [21]. Hair removal methods such as laser therapy and electrolysis were commonly used for nonsurgical gender-affirming purposes. Interestingly, a single case report described complete scalp hair regrowth in a transgender woman receiving estrogen therapy [22]. Overall, dermatologic manifestations appear more prominent in transmasculine individuals, while persistent hair growth remains a major issue in transfeminine and nonbinary populations.

### 3.3. Gynecologic Effects

The gynecologic effects of GAHT were assessed in several prospective and retrospective studies (Supplementary Table S3). Histological analyses of transmasculine individuals on long-term testosterone showed active or secretory endometrium in some cases, with multifollicular ovaries frequently present [23]. Other series reported predominantly proliferative or atrophic endometrial patterns in hysterectomy specimens from transmasculine individuals [24]. Ovarian cortical follicle distribution in testosterone users was similar to that of fertile cisgender women, although the implications of these histological findings for subsequent fertility remain uncertain [25], while polycystic ovarian morphology was not increased compared with controls [26]. A novel epithelial prostatic glandular proliferation in the vaginal epithelium was described in transmasculine individuals under long-term androgen exposure [27]. Amenorrhea was typically achieved within three months of testosterone initiation, particularly in those with higher hemoglobin levels [28]. Qualitative studies highlighted knowledge gaps among both patients and providers regarding the impact of testosterone on pregnancy risk and contraceptive choices, with condoms being the main method used [29]. In transfeminine individuals, sustained breast development was documented over three years of feminizing therapy, with most reporting satisfaction with breast size [30].

### 3.4. Musculoskeletal Effects

Musculoskeletal outcomes of GAHT showed consistent anabolic effects in transmasculine individuals and more heterogeneous findings in transfeminine individuals (Supplementary Table S4). Several longitudinal and cross-sectional studies demonstrated significant increases in muscle mass, strength, and lean body mass in transmasculine individuals, with concomitant reductions in fat mass and largely stable bone mineral density [31,32,33]. Long-term androgen exposure was associated with bone mineral density values comparable to cisgender men and higher than cisgender women [34]. In contrast, transfeminine individuals often exhibited lower baseline bone mineral density and lean mass, with increases in fat mass and stable or modestly reduced bone parameters over time [35,36]. Studies in adolescents highlighted the impact of gonadotropin-releasing hormone agonist (GnRHa) on reduced bone accrual and growth velocity. Subsequent GAHT was associated with partial recovery of bone mineral accrual, although bone mineral density z-scores did not consistently normalize, particularly in transfeminine adolescents [37,38]. Progestins (cyproterone acetate or lynestrenol) also influenced body composition changes consistent with affirmed gender appearance [39]. Notably, failure to achieve expected body composition changes was linked to inadequate testosterone levels or insufficient LH suppression [40]. Overall, GAHT supports musculoskeletal adaptation aligned with affirmed gender, but bone health requires close monitoring, especially in transfeminine and adolescent populations using GnRH agonists. While bone health in transmasculine individuals is generally preserved across studies, those with risk factors for bone disease should still be appropriately monitored.

### 3.5. Biological and Pharmacological Effects

The biological effects of GAHT encompass hematologic, metabolic, endocrine, and reproductive domains (Supplementary Table S5). Hematologic changes included increased hemoglobin, hematocrit, and creatinine in transmasculine individuals, with opposite trends in transfeminine individuals, stabilizing within six months of therapy initiation [41,42]. Erythrocytosis was frequently observed in long-term testosterone users, influenced by smoking, body mass index (BMI), and administration route [43]. Endocrine alterations comprised suppressed antimüllerian hormone in transgender men [44] and effective testosterone suppression with both cyproterone acetate and leuprolide regimens in trans women [45,46]. Estradiol pharmacokinetics varied by formulation, with injectable preparations achieving higher concentrations than oral or transdermal routes [47]. Metabolic changes included shifts in lipid fractions, insulin sensitivity, and inflammatory markers, with masculinization associated with increased insulin sensitivity and feminization with reductions [48,49]. Hypercoagulable parameters were demonstrated in transfeminine individuals, although with increased fibrinolytic potential [50]. Drug–drug interactions were reported between feminizing therapy and antiretrovirals, reducing tenofovir exposure [51,52]. Fertility implications included reduced sperm quality in trans women exposed to GAHT [53]. Overall, GAHT induced predictable shifts in biological markers, with clinical relevance for monitoring safety, tailoring regimens, and guiding fertility preservation counseling.

### 3.6. Psychiatric Effects

Psychiatric outcomes of GAHT consistently suggest improvements in mental health and quality of life (Supplementary Table S6). Prospective studies suggest that gender-affirming hormone therapy improves mental health outcomes in transgender individuals. Evidence indicates early reductions in gender dysphoria and improvements in quality of life, with longer-term follow-up also showing decreases in symptoms of depression and anxiety [54,55]. Cross-sectional evidence indicated that transgender individuals not receiving hormones had a nearly fourfold higher risk of probable depressive disorder compared with cisgender controls, whereas cross-sex hormone treatment (CHT) use was protective [56]. GAHT was also associated with lower rates of eating disorder symptoms [57] and reduced suicidality in transgender youth [58]. Access to pubertal suppression during adolescence was also associated with lower lifetime suicidal ideation in adulthood [59]. Personality-related changes included decreased neuroticism and increased extraversion after testosterone initiation in transmasculine individuals [60], along with greater psychological functioning across multiple domains [61]. Case reports described rare psychiatric complications, such as estrogen withdrawal-related psychosis [62]. One study reported that sexual desire and arousal increased with testosterone in transmasculine individuals, though mood measures remained stable [63]. Overall, GAHT appears to improve psychiatric outcomes, with reductions in depression, suicidality, and dysphoria, although rare adverse effects warrant attention.

### 3.7. Other Side Effects and Specific Clinical Situations

Beyond cardiometabolic, dermatologic, gynecologic, musculoskeletal, biological, and psychiatric domains, several other adverse events and specific clinical scenarios have been reported (Supplementary Table S7). Case reports described the development or progression of meningiomas in transfeminine individuals after long-term estrogen therapy [64,65]. Rare oncologic events were noted, including breast cancer in a transmasculine individual on long-term testosterone [66] and metastatic cancer in a trans woman shortly after estrogen initiation [67]. Cases of autoimmune diseases such as lupus nephritis and systemic lupus erythematosus in transfeminine individuals [68,69] and rheumatoid arthritis in a transmasculine individual [70] have also been reported, while testosterone was linked to clinical improvement in cutaneous lupus [71]. Pharmacovigilance data identified only a small number of suspected adverse drug reactions to GAHT, including pancreatitis, gynecomastia, musculoskeletal complaints, and bleeding events [5]. A web-based survey suggested a higher COVID-19 infection rate among transmasculine than transfeminine individuals [72]. Acoustic voice variables in transgender men did not differ significantly from cisgender male controls despite testosterone exposure [73]. Other isolated cases included pseudoangiomatous stromal hyperplasia in a transfeminine individual [74] and positive psychosocial outcomes of pubertal suppression with GnRHa in a non-binary adolescent [75].

## 4. Discussion

Our review highlights the wide range of treatment-related issues addressed in the medical literature concerning transgender individuals. These topics represent critical dimensions to consider when designing the content of pharmacist-led consultations with transgender individuals in community pharmacies.

Various approaches have been proposed to assess the information needs of transgender individuals. However, there is currently no dedicated tool specifically designed for this population. Most instruments assess general information needs, with limited specificity for particular clinical contexts. Consequently, we opted to conduct a narrative synthesis of treatment-related challenges in transgender care. This approach should be complemented by further research directly involving transgender individuals.

The following practical implications are based on the findings of the included studies and, where applicable, current clinical guidelines. Proposed pharmacist roles reflect the authors’ interpretation of how these findings may inform community pharmacy practice.

The synthesis of the current evidence highlights that gender-affirming hormone therapy is associated with a broad spectrum of cardiovascular, dermatologic, gynecologic, musculoskeletal, biological, and psychiatric effects. These findings carry important implications for community pharmacists, who are often the most accessible healthcare professionals for transgender individuals and thus play a central role in monitoring, counseling, and optimizing therapy. From a cardiovascular perspective, observational studies have reported associations between GAHT and venous thromboembolism, stroke, and changes in lipid profiles, particularly among transfeminine individuals, while transmasculine populations may experience obesity and atherogenic lipid changes. However, these findings should be interpreted cautiously, as the observational nature of much of the available evidence limits the ability to establish causal relationships. Pharmacovigilance data should therefore be interpreted as safety signals rather than as estimates of adverse event incidence. The current evidence on endothelial function in transmasculine individuals remains limited and inconsistent [16,76]. Community pharmacists should therefore reinforce adherence to monitoring schedules, provide lifestyle counseling on modifiable risk factors, and encourage early medical consultation when warning symptoms occur. Dermatologic manifestations such as acne and alopecia in transmasculine individuals and persistent hair growth in transfeminine individuals remain frequent sources of distress; community pharmacists can provide appropriate counselling on these treatment-related issues, including information on available hair-removal options and suitable over-the-counter treatments within their scope of practice, with referral to dermatology when further assessment is required. The available evidence on sexual health indicates that testosterone influences sexual function in transmasculine individuals, particularly sexual desire, sexual arousal, and pain or discomfort during sexual activity with recent evidence highlighting growing attention to this topic [77,78]. Gynecologic data underline the importance of fertility preservation discussions, amenorrhea monitoring, and contraceptive counseling. Pharmacists can contribute by ensuring that patients receive accurate information about pregnancy risks under testosterone therapy and safe contraceptive options, addressing well-documented knowledge gaps for both patients and providers. These findings are consistent with those of a recent review of testosterone-based gender-affirming hormone therapy, which emphasized the diverse effects of testosterone on gynecologic function in transmasculine individuals, including several underexplored outcomes, and concluded that care should be individualized and gender-affirming [79].

Biological and pharmacological findings further emphasize the need for systematic laboratory follow-up. Community pharmacists can rely on standard monitoring plans based on international recommendations [80]. Hematologic shifts, erythrocytosis, and metabolic disturbances highlight the importance of counseling on smoking cessation, weight management, and safer administration routes. Moreover, drug–drug interactions, notably with antiretrovirals, require pharmacists to maintain vigilance in HIV-positive transgender women and those using pre-exposure prophylaxis. Musculoskeletal outcomes, particularly reduced bone accrual in adolescents undergoing GnRHa therapy and decreased bone density in transfeminine adults, highlight treatment-related issues that may warrant counselling on vitamin D and calcium intake, lifestyle measures, and adherence to recommended follow-up, as well as referral when further clinical assessment is indicated.

Psychiatric outcomes are encouraging, with reductions in depression, suicidality, and dysphoria observed under GAHT. However, these findings should be interpreted cautiously, as much of the available evidence is observational. A recent study showed that GAHT was associated with improvements in quality of life as early as 3 months after the initiation of testosterone therapy [77]. These findings underscore the pharmacist’s role not only as a medication expert but also as a supportive healthcare provider who can recognize mental health concerns, provide empathetic listening, and facilitate timely referral to psychological services. Finally, while rare, serious adverse events such as meningiomas, autoimmune conditions, and hormone-related malignancies highlight the need for vigilance, patient education, and collaboration with prescribers to ensure early recognition and management.

Although the WPATH Standards of Care Version 8 (SOC-8), do not define a specific role for community pharmacists, they emphasize individualized, multidisciplinary care and collaboration among healthcare professionals involved in transgender health [1]. This multidisciplinary framework provides a basis for considering how community pharmacists could contribute to medication-related care within their scope of practice, particularly through medication counseling, treatment monitoring, identification of potential adverse effects and drug–drug interactions, and referral to other healthcare professionals when appropriate. The literature provides substantial information on the clinical and pharmacological effects of GAHT, but there is limited evidence on how this information can be systematically translated into community pharmacy practice. In particular, the available literature does not establish how community pharmacists should structure medication reviews, monitoring, counseling, identification of treatment-related risks, and referral within multidisciplinary transgender care pathways. This represents an important area for future research and service development.

Several international frameworks recognize the pharmacist’s contribution to patient care and interprofessional collaboration. The joint guidelines developed by the International Pharmaceutical Federation (FIP) and the World Health Organization (WHO) provide internationally recognized standards for pharmaceutical services. These guidelines stress the importance of personalized counseling, monitoring treatment adherence, and promoting public health [81]. Furthermore, the Council of Europe resolution on pharmaceutical care advocates for integrating pharmacists into national health policies and care teams, recognizing their essential role in optimizing medication use [82]. It particularly recommends involving pharmacists in multidisciplinary teams and fostering interprofessional coordination. Although these frameworks are not specific to transgender healthcare, they provide a general professional framework that can be adapted to the medication-related needs identified in our review.

A number of literature reviews have addressed the evolving role of pharmacists in transgender health care [3,4,83]. These reviews emphasize that pharmacists can provide counseling on hormone therapy with particular attention to self-medication, monitoring drug–drug interactions, supporting patients in managing hormone-related side effects, and offering a welcoming, inclusive environment through the use of appropriate language. This approach may contribute to patient-centered and equitable care for transgender individuals, although its effects on therapeutic outcomes, patient satisfaction, and healthcare disparities have not been directly evaluated in the studies included in this review. The scope of community pharmacy practice varies across countries and healthcare systems. Accordingly, the potential pharmacist roles described below should be interpreted within the relevant national regulatory and professional frameworks, with referral to physicians or other healthcare professionals when assessment or management falls outside the pharmacist’s scope of practice.

The expanding role of community pharmacists in transgender care requires specific education and training to understand the pharmacological implications of gender-affirming therapies and to deliver inclusive, patient-centered care supported by appropriate educational resources [84,85]. Such training may be provided through undergraduate education, continuing professional development, or other accredited educational programs, according to national frameworks. The implementation of such consultations may also depend on organizational, relational, and economic factors, including privacy, continuity of care, access to relevant clinical and laboratory information, coordination with other healthcare professionals, trust with transgender individuals, and affordability and reimbursement of gender-affirming treatments. Particular consideration should be given to adolescents receiving GnRHa or GAHT. In this population, structured pharmacist-led consultations should take into account age-specific developmental and monitoring needs, while respecting adolescents’ autonomy, privacy, and applicable requirements regarding consent and confidentiality. When appropriate and in accordance with applicable legal and institutional frameworks, parents or caregivers may also be involved in the consultation and support of treatment. Close coordination with the prescribing and specialist care team is particularly important when clinical assessment or treatment-related decisions are required.

This review has several limitations. First, the systematic literature search was conducted on 17 July 2024, and was not subsequently updated before manuscript completion. Given the rapidly evolving literature on transgender healthcare, relevant studies published after this date may therefore not have been captured by the systematic search. The literature search was conducted in MEDLINE, Google Scholar, and APA PsycINFO, but did not include Embase or Web of Science, which may have resulted in the omission of some relevant biomedical or pharmacological literature. In addition, the use of the “allintitle:” operator for Google Scholar and the restriction of this search to publications from 2012 onwards may have reduced the sensitivity of this information source. Of the 201 reports sought for retrieval after title and abstract screening, 43 could not be obtained for full-text assessment. Although attempts were made to retrieve these reports through the available electronic and institutional access, their exclusion may have introduced selection bias if relevant eligible evidence was contained in these inaccessible publications. This limitation should be considered when interpreting the comprehensiveness of the evidence identified by the review. Although the literature search was conducted according to the PRISMA methodology, the included studies were highly heterogeneous in terms of study design, participant characteristics (including age), gender-affirming hormone therapy regimens (including the specific hormones used, routes of administration, dosing regimens, concomitant antiandrogens or GnRH analogues, and duration of treatment), outcomes assessed, and duration of follow-up, limiting direct comparisons across studies. Second, much of the available evidence consisted of observational studies, retrospective analyses, and case reports, whereas prospective studies remained limited for several clinical outcomes. Third, no formal risk-of-bias assessment was performed because the primary objective of this review was not to estimate the magnitude of treatment effects but to identify clinically relevant treatment-related issues that may be relevant to pharmacist-led consultations with transgender individuals. Nevertheless, the heterogeneity of the included study designs limits the ability to formally compare the methodological quality and strength of evidence across studies. Findings were therefore interpreted according to the nature of the available evidence, with observational studies, pharmacovigilance reports, and case reports not considered equivalent in terms of evidentiary strength. The protocol was registered retrospectively in PROSPERO after completion of the initial screening and data extraction, which limits the ability to demonstrate that the eligibility criteria, classification domains, and methodological decisions were established before the results were known. No substantive deviations from the registered protocol were identified. Finally, the available literature mainly focused on gender-affirming hormone therapy, whereas other aspects of pharmaceutical care, such as self-medication practices, medication adherence, patients’ expectations, and information needs, remain insufficiently investigated.

## 5. Conclusions

This review identifies the key treatment-related issues that may inform the development of future pharmacist-led consultations with transgender individuals in community pharmacy practice. The findings provide an evidence-informed basis for developing structured pharmaceutical consultations that integrate clinical monitoring, optimization of pharmacotherapy, prevention and management of treatment-related adverse effects, patient-centered communication, and inclusive practices. They also highlight the need for dedicated education and training to equip pharmacists with the knowledge and skills required to provide evidence-based, gender-affirming care and to contribute effectively to multidisciplinary care for transgender individuals. Future consultation frameworks should be co-designed with transgender individuals and relevant healthcare professionals and require validation and evaluation of their feasibility, acceptability, and effectiveness in clinical practice.

## Data Availability

The original contributions presented in this study are included in the article/Supplementary Materials. Further inquiries can be directed to the corresponding author.

## Supplementary Materials

The following supporting information can be downloaded at: https://www.mdpi.com/article/10.3390/pharmacy14060132/s1, File S1: Complete search strategies for Medline, APA PsycINFO and Google Scholar; Table S1: Studies reporting data on cardiovascular effects and cardiometabolic risks; Table S2: Studies reporting data on dermatologic effects; Table S3: Studies reporting data on gynecologic effects; Table S4: Studies reporting data on musculoskeletal effects; Table S5: Studies reporting data on biological effects (*:effects potentially related to drug–drug interactions); Table S6: Studies reporting data on psychiatric effects; Table S7: Studies reporting data on other side effects and specific clinical situation. Table S8: Summary of treatment-related issues associated with GAHT and their potential relevance to community pharmacy practice [86,87,88,89,90,91,92,93,94,95,96,97,98,99,100,101,102,103,104,105,106,107,108,109,110,111,112,113,114,115,116,117,118,119,120,121,122,123,124,125,126,127,128,129,130,131].
