# Supplementary material for: Therapeutic Issues Associated with Gender-Affirming Hormone Therapy in Transgender Individuals: A Systematic Review Relevant to Community Pharmacy Practice
Source: Pharmacy (Basel). 2026 Sep 10;14(6):132. doi: 10.3390/pharmacy14060132 (PMC13610454; doi:10.3390/pharmacy14060132)
Supplement: Supplementary file 1 [file pharmacy-14-00132-s001.zip › Supplementary File S1.pdf]

Supplementary File S1 – Complete search strategies for Medline, APA PsycINFO and Google Scholar

MEDLINE
Database/interface : MEDLINE via PubMed
Date of search : 17 July 2024
Date limits : No restriction
Language limits : None applied at database-search stage
Search strategy :
((((Transsexual[Title/Abstract]) OR (Transgender[Title/Abstract])) OR (Transgender people[Title/Abstract])) OR (Transgender Persons[Title/Abstract]) OR (((transgender[MeSH Terms]) OR (transgenders[MeSH Terms])) OR (transsexualism[MeSH Terms])) OR (transsexualisms[MeSH Terms])) AND ((((((((Adverses drug reaction[Title/Abstract]) OR (Access to care[Title/Abstract])) OR (community health[Title/Abstract])) OR (underserved population[Title/Abstract])) OR (pharmacist[Title/Abstract])) OR (Preventive care[Title/Abstract])) OR (Preventive healthcare[Title/Abstract])) OR (Health care for transgender people[Title/Abstract]) OR (((((((((drug therapy[MeSH Terms]) OR (adverse drug event*[MeSH Terms])) OR (adverse drug reaction*[MeSH Terms])) OR (drug interaction*[MeSH Terms])) OR (health facility[MeSH Terms])) OR (health care delivery[MeSH Terms])) OR (minority health[MeSH Terms])) OR (healthcare disparit*[MeSH Terms])) OR (cardiovascular disease*[MeSH Terms])) OR (body composition*[MeSH Terms])) AND (((androgen*[MeSH Terms]) OR (estrogen*[MeSH Terms])) OR (testosteron*[MeSH Terms]) OR (((((hormone therapy[Title/Abstract]) OR (hormonal replacement therapy[Title/Abstract])) OR (sex steroid hormone*[Title/Abstract])) OR (Testosterone[Title/Abstract])) OR (estrogen*[Title/Abstract])) OR (Gender affirming treatment[Title/Abstract])).

APA PsycINFO
Database/interface: APA PsycINFO via EBSCOhost
Database: APA PsycINFO (PsycINFO)
Date of search: 17 July 2024
Search terms: transgender AND hormone therapy
Expansion option: Apply equivalent subjects
Source type: Academic journals
Publication date: No restriction
Search strategy:
transgender AND "hormone therapy"

Google Scholar
Database/interface: Google Scholar
Date of search: 17 July 2024
Search field: allintitle:
Publication date limit: Since 2012
Search procedure: Multiple title-based searches were performed using combinations of transgender-related terms and hormone-therapy-related terms:

Search string	Publication date limit	Results retrieved
allintitle: transsexual "hormone therapy"	Since 2012	1
allintitle: transsexual "drug for cross gender hormonal replacement therapy"	Since 2012	0
allintitle: transsexual "drug for irreversible hormonal replacement therapy"	Since 2012	0
allintitle: transsexual "sex steroid hormones"	Since 2012	0

Search string	Publication date limit	Results retrieved
allintitle: transsexual "testosterone"	Since 2012	0
allintitle: transsexual "oestrogens"	Since 2012	0
allintitle: transsexual "transgender men"	Since 2012	0
allintitle: transsexual "transgender women"	Since 2012	0
allintitle: transsexual "gender affirming treatment"	Since 2012	0
allintitle: Transgender "hormone therapy"	Since 2012	48
allintitle: Transgender "Drug for cross gender hormonal replacement therapy"	Since 2012	0
allintitle: Transgender "drug for irreversible hormonal replacement therapy"	Since 2012	0
allintitle: Transgender "sex steroid hormones"	Since 2012	0
allintitle: Transgender "oestrogens"	Since 2012	1
allintitle: Transgender "gender affirming treatment"	Since 2012	5
allintitle: Transgender people "hormone therapy"	Since 2012	6
allintitle: Transgender people "drug for cross gender hormonal replacement therapy"	Since 2012	0
allintitle: Transgender people "drug for irreversible hormonal replacement therapy"	Since 2012	0
allintitle: Transgender people "sex steroid hormones"	Since 2012	0
allintitle: Transgender people "testosterone"	Since 2012	0
allintitle: Transgender people "oestrogens"	Since 2012	0
allintitle: Transgender people "gender affirming treatment"	Since 2012	0
allintitle: transgender persons "hormone therapy"	Since 2012	1
allintitle: transgender persons "drug for cross gender hormonal replacement therapy"	Since 2012	0
allintitle: transgender persons "drug for irreversible hormonal replacement therapy"	Since 2012	0
allintitle: transgender persons "sex steroid hormones"	Since 2012	0
allintitle: transgender persons "testosterone"	Since 2012	0

Search string	Publication date limit	Results retrieved
allintitle: transgender persons "oestogens"	Since 2012	0
allintitle: transgender persons "transgender men"	Since 2012	2
allintitle: transgender persons "transgender women"	Since 2012	2
allintitle: transgender persons "gender affirming treatment"	Since 2012	0
**Total**		**66**
**After duplicate removal**		**57**

The Google Scholar search was conducted using the “allintitle:” operator and was limited to publications from 2012 onwards. All 66 retrieved results were screened for eligibility. No formal stopping criterion was prespecified. After removal of duplicates, 57 articles remained for screening.
