# Supplementary material for: Therapeutic Issues Associated with Gender-Affirming Hormone Therapy in Transgender Individuals: A Systematic Review Relevant to Community Pharmacy Practice
Source: Pharmacy (Basel). 2026 Sep 10;14(6):132. doi: 10.3390/pharmacy14060132 (PMC13610454; doi:10.3390/pharmacy14060132)
Supplement: Supplementary file 1 [file pharmacy-14-00132-s001.zip › Supplementary Table S1.pdf]

Supplementary Table S1. Studies reporting data on cardiovascular effects and cardiometabolic risks

Reference (year)	Transgender people concerned	Study type	Objectives	Clinical data to consider
**Getahun et al. (2018)**[8]	Transmasculine and transfeminine individuals	Retrospective cohort study based on electronic medical records	To examine the incidence of venous thromboembolism, ischemic stroke, and myocardial infarction events in a cohort of transgender persons	A significantly higher incidence of venous thromboembolism was observed among transfeminine individuals compared with cisgender men and cisgender women, whereas the incidence of ischemic stroke and myocardial infarction did not differ across groups.
**Mullins et al. (2021)**[11]	Adolescent and young adult transgender population	Retrospective chart review	To examine thrombosis and thrombosis risk factors among an exclusively adolescent and young adult transgender population	No arterial or venous thrombotic events associated with gender-affirming hormone therapy were identified in transgender women receiving estrogen or transgender men receiving testosterone.
**Karalexi et al. (2022)**[10]	Transmasculine and transfeminine individuals	Population-based cohort study	To compare the incidence rates of cardiovascular outcomes in transgender individuals with and without GAHT to the rates in cisgender individuals from the general population	Transgender individuals had higher cardiovascular disease incidence rates than cisgender controls regardless of GAHT exposure; however, absolute risks were low and events were predominantly benign arrhythmias.
**Yelehe et al (2022)**[6]	Transmasculine and transfeminine individuals	Nationwide retrospective study	To analyze ADRs related to gender-affirming hormone therapy reported in the French pharmacovigilance database	Among transgender women, meningiomas were the most frequently reported adverse drug reactions, followed by cardiovascular events. In transgender men receiving testosterone enanthate, all reported adverse drug reactions were cardiovascular, with pulmonary embolism accounting for half of the cases.
**Balcerek et al (2021)**[86]	Transfeminine individuals	Retrospective cross-sectional analysis	To evaluate feminizing hormone therapy regimens and cardiovascular risk factors in aging trans individuals	Transdermal estradiol was more frequently prescribed in transgender women aged ≥45 years; among those receiving oral estradiol, the median daily dose was lower.
**Cocchetti et al (2021)**[12]	Transmasculine and transfeminine individuals	Multicenter prospective cohort study	To evaluate changes in the 30-year Framingham cardiovascular disease risk in a large cohort of transgender individuals after the start of GAHT	In transgender women, triglycerides, total cholesterol, and LDL cholesterol levels decreased significantly during two years of follow-up. In contrast, transgender men experienced unfavorable lipid changes, including increased total cholesterol, triglycerides, and LDL cholesterol levels, along with decreased HDL cholesterol.
**Hirsch et al (2022)**[14]	Transfeminine individuals	Case report	Presentation of a clinical case	A case of spontaneous coronary artery dissection was described in a young transgender woman after nine years of continuous GAHT.

**Gulanski et al (2020)**[16]	Transmasculine individuals	Prospective observational study	To determine the effect of testosterone treatment on endothelial function in transgender men.	Transgender men had higher total and free testosterone concentrations than cisgender women and exhibited significantly reduced flow-mediated vasodilation, indicating impaired endothelial function.
**Kozato et al (2021)**[87]	Transgender and gender nonbinary people	Single-center retrospective chart review	To examine the venous thromboembolism risk and incidence associated with suspension of hormone therapy versus its continuation in the perioperative period for gender-affirming surgeries	Only one venous thromboembolism event occurred during the study period, and no events were observed among patients who continued hormone therapy perioperatively.
**Fan et al (2020)**[88]	Transmasculine individuals	Case report	Presentation of a clinical case	A 17-year-old transgender man without known inherited thrombophilia developed a pulmonary embolism while receiving testosterone injections for gender dysphoria.
**Ciccone et al (2017)**[89]	Transmasculine and transfeminine individuals	Population-based, matched cohort study	To evaluate and stratify early cardiovascular risk of transsexuals who underwent pharmacological and/or surgical gender reassignment	Markers of endothelial and morphological dysfunction suggested increased early cardiovascular risk in individuals who underwent gonadectomy compared with those receiving cross-sex hormone therapy alone.
**Stanley et al (2018)**[90]	Transgender adolescent	Case report	Presentation of a clinical case	A pulmonary embolism occurred in a transgender adolescent receiving both estrogen and testosterone therapy, in the absence of an identified genetic thrombophilic condition.
**Andzembe et al (2023)**[15]	Transfeminine individual	Case report	Presentation of a clinical case	A case of branch retinal vein occlusion with associated visual loss was reported in a 44-year-old transgender woman undergoing long-term hormone replacement therapy.
**Nota et al (2019)**[9]	Transmasculine and transfeminine individuals	Retrospective cohort study	To determine the incidences of acute/spontaneous strokes, myocardial infarctions, and venous thromboembolic events in transgender men and women receiving transgender hormone therapy	Transgender women had higher adjusted incidence rates of stroke and venous thromboembolism than reference populations, and both transgender women and men had an increased risk of myocardial infarction.
**Martinez-Martin et al (2023)**[91]	Transmasculine and transfeminine individuals	Retrospective cohort study	To assess the risk of hypertension development	Although a causal relationship could not be definitively established, cyproterone acetate use was associated with an increased risk of hypertension, suggesting that its use for this indication should be reconsidered.
**Arnold et al (2016)**[92]	Transfeminine individuals	Retrospective chart review	To estimate the incidence of venous thromboembolism events from the standard feminizing cross-sex hormone therapy used in the United States	Venous thromboembolism occurred in 0.15% of transgender women receiving feminizing hormone therapy, corresponding to an incidence of 7.8 events per 10,000 person-years.

**Wierckx et al (2013)**[118]	Transmasculine and transfeminine individuals	Cross-sectional study	To evaluate the short- and long-term cardiovascular- and cancer-related morbidities during cross-sex hormone therapy	Transgender women experienced more myocardial infarctions than cisgender women but a similar proportion compared with cisgender men. Cerebrovascular disease was more prevalent in transgender women than in cisgender men, while rates in transgender men were comparable to those of cisgender controls.
**LaHue et al (2019)**[94]	Transfeminine individuals	Cohort study	To describe the clinical characteristics and risk factors of transgender women	Ischemic stroke was the most frequently observed cerebrovascular event, followed by intracerebral hemorrhage, transient ischemic attack, and combined ischemic stroke with subarachnoid hemorrhage
**Choy et al (2022)**[95]	Transmasculine individual	Case report	Presentation of a clinical case	A case of branch retinal artery occlusion was reported in a transgender man receiving weekly testosterone injections.
**Wierckx et al (2014)**[93]	Transfeminine individual	Case report	Presentation of a clinical case	A transgender woman developed acute painless bilateral vision loss during cross-sex hormone therapy.
**Park et al (2014)**[96]	Transmasculine individual	Case report	Presentation of a clinical case	A case of intracranial hypertension was reported in a transgender man receiving testosterone therapy.
**Chan et al (2017)**[97]	Transfeminine individual	Case report	Presentation of a clinical case	A deep vein thrombosis was reported in a transgender woman undergoing feminizing hormone therapy.
**Park et al (2019)**[98]	Transmasculine individual	Case report	Presentation of a clinical case	A spontaneous superior ophthalmic vein thrombosis was reported in a transgender man with systemic lupus erythematosus.
**Vita et al. (2018)**[99]	Transmasculine and transfeminine individuals	Retrospective cohort study	To assess the hormonal and metabolic effects of cross-sex hormone therapy	In transgender women, systolic blood pressure, red blood cell count, hemoglobin, hematocrit, and total testosterone levels decreased significantly compared with baseline values.
**Fernandez et al (2016)**[100]	Transmasculine and transfeminine individuals	Retrospective chart review	To determine if transgender hormone therapy with estrogen and spironolactone for transgender women or with testosterone for transgender men had adverse anthropomorphic or metabolic effects	No significant changes in blood pressure were observed in either transgender women or men. Body mass index increased among transgender men, and HDL cholesterol increased among transgender women.
**Nokoff et al (2020)**[101]	Transmasculine and transfeminine individuals	Cross-sectional study	To determine insulin sensitivity and body composition among transgender women and men on estradiol or testosterone, compared with cisgender women and men	Transgender adolescents receiving gender-affirming hormone therapy exhibited body composition profiles intermediate between BMI-matched cisgender males and females, and transgender women demonstrated lower insulin sensitivity than cisgender men.

**van Velzen et al (2021)**[102]	Transmasculine and transfeminine individuals	Cross-sectional study based on a random sample from the European Network for the Investigation of Gender Incongruence	To investigate the effects of transgender HT treatment on cholesterol efflux capacity (HDL-CEC)	In transgender women, hormone therapy significantly reduced total HDL cholesterol efflux capacity and ABCA1-mediated HDL efflux.
**Deutsch et al (2015)**[103]	Transmasculine and transfeminine individuals	Prospective cohort study	To describe weight, body mass index, blood pressure, lipids, and hormone levels in transgender women and men presenting for initiation of cross-sex hormone therapy at a community clinic in the United States	Median blood pressure and lipid values were within the normal range before and after six months of cross-sex hormone therapy in a mixed cohort of transgender women and men.
**van Velzen et al (2019)**[13]	Transmasculine and transfeminine individuals	Prospective, observational study	To assess the effects of 1 year of treatment with oral or transdermal administration of estrogen (plus cyproterone) and transdermal or IM application of testosterone on serum lipid levels and blood pressure	In transgender men, hormone therapy was associated with increases in diastolic blood pressure, total cholesterol, low-density lipoprotein cholesterol, and triglycerides, along with a decrease in high-density lipoprotein cholesterol. In transgender women, slight decreases in blood pressure and reductions in total cholesterol, LDL cholesterol, HDL cholesterol, and triglycerides were observed.
**Millington et al (2021)**[104]	Transmasculine adolescents	Cross-sectional study	To examine lipoprotein profiles in individuals with XX chromosomes treated with testosterone	Transmasculine adolescents exhibited lipoprotein profiles similar to those of cisgender males and more atherogenic than those of cisgender females, characterized by higher concentrations of small LDL particles and lower concentrations of large HDL particles.
**Valentine et al (2022)**[105]	Transgender and gender-diverse youth	Multicenter, retrospective, cross-sectional study	To determine the odds of cardiometabolic-related diagnoses in transgender and gender-diverse youth (TGDY) compared to matched controls in a cross-sectional analysis	Transgender and gender-diverse youth had higher odds of overweight and obesity compared with matched controls. Those prescribed testosterone, alone or in combination with gonadotropin-releasing hormone agonists, had higher odds of dyslipidemia and liver dysfunction than those not receiving gender-affirming hormone therapy.
**Sequeira et al (2019)**[17]	Transmasculine adolescents	Retrospective chart review	To determine the effect of testosterone on body mass index (BMI) z-score in transmasculine adolescents at 6 and 12 months after initiation	Body mass index z-scores increased significantly in transmasculine adolescents after six months of testosterone therapy, with no significant difference observed between baseline and 12 months.
**Klaver et al (2022)**[106]	Transmasculine and transfeminine individuals	Prospective cross-sectional study	To investigate changes in visceral fat and the ratio of visceral fat to total body fat (VAT/TBF) and their associations with changes in lipids and	Hormone therapy resulted in only small mean changes in visceral fat. Changes in the ratio of visceral to total body fat were not associated with changes in lipid levels or insulin

			insulin resistance after 1 year of hormone therapy in trans persons	resistance in either transgender women or men after one year of treatment.
**Quiros et al (2015)**[107]	Transmasculine and transfeminine individuals	Retrospective longitudinal study	To assess baseline differences in lipid profile in individuals with gender identity disorder in relation to prior CHT, and changes in the lipid profile and other cardiovascular risk factors after 24 months of treatment	During follow-up, transgender women experienced significant increases in body weight and body mass index, as well as increases in systolic and diastolic blood pressure, although values remained within the normal range. No significant changes in lipid profiles were observed. Transgender men also showed increases in body weight and body mass index without significant changes in blood pressure.
**Fung et al (2016)**[108]	Transfeminine individuals	Retrospective chart review	To compare the effects of spironolactone and cyproterone acetate on HDL and prolactin serum concentrations in transgender women.	Spironolactone use in transgender women was associated with increased HDL cholesterol levels, whereas cyproterone acetate was associated with reduced HDL cholesterol and higher prolactin concentrations.
**Kwan et al (2019)**[109]	Transfeminine individual	Case report	Presentation of a clinical case	A case of cerebellar infarction was reported in a transgender woman with extensive intracranial and extracranial atherosclerosis identified on imaging.
**Kyinn et al (2021)**[18]	Transmasculine and transfeminine individuals	Longitudinal study	To assess weight gain and obesity rates in transgender and gender-diverse adults before and during hormone therapy	Transmasculine individuals exhibited greater rates of obesity and weight gain before and during hormone therapy compared with transfeminine individuals.
