# Supplementary material for: Therapeutic Issues Associated with Gender-Affirming Hormone Therapy in Transgender Individuals: A Systematic Review Relevant to Community Pharmacy Practice
Source: Pharmacy (Basel). 2026 Sep 10;14(6):132. doi: 10.3390/pharmacy14060132 (PMC13610454; doi:10.3390/pharmacy14060132)
Supplement: Supplementary file 1 [file pharmacy-14-00132-s001.zip › Supplementary Table S2.pdf]

Supplementary Table S2. Studies reporting data on dermatologic effects

**Reference (year)**	**Transgender people concerned**	**Study type**	**Objectives**	**Clinical data to consider**
**Thoreson et al (2021)**[19]	Transmasculine individuals	Retrospective cohort study	To assess risk of acne among a large population of transgender patients receiving masculinizing hormone therapy and clinical risk factors for acne diagnosis	The overall prevalence of acne increased from 6.3% before initiation of masculinizing hormone therapy to 31.1% after a mean treatment duration of 3.4 years. Individuals aged 18–21 years appeared to be at highest risk.
**Marks et al (2020)**[21]	Transfeminine individuals and nonbinary people	Cross-sectional survey study	To identify the frequency of unwanted facial and body hair, the use of various hair removal methods, and associated barriers to care in gender minority patients	Excess facial and/or body hair was reported by 84% of transgender women receiving feminizing hormone therapy, 100% of transgender women not receiving therapy, and 100% of nonbinary individuals receiving feminizing therapy. Laser hair removal and electrolysis were commonly used for gender-affirming purposes.
**Thoreson et al (2021)**[20]	Transmasculine individuals	Retrospective cohort study	To examines the proportion of transgender and gender-diverse individuals patients receiving masculinizing hormone therapy who developed androgenetic alopecia	The prevalence of androgenetic alopecia increased from 0.4% before masculinizing hormone therapy to 3.1% after initiation, with the highest incidence observed during the fourth year of therapy.
**Adenuga et al (2012)**[22]	Transfeminine individuals	Case report	Presentation of a clinical case	Complete scalp hair regrowth was reported in a transgender woman receiving estrogen therapy.
