# Supplementary material for: Therapeutic Issues Associated with Gender-Affirming Hormone Therapy in Transgender Individuals: A Systematic Review Relevant to Community Pharmacy Practice
Source: Pharmacy (Basel). 2026 Sep 10;14(6):132. doi: 10.3390/pharmacy14060132 (PMC13610454; doi:10.3390/pharmacy14060132)
Supplement: Supplementary file 1 [file pharmacy-14-00132-s001.zip › Supplementary Table S3.pdf]

Supplementary Table S3. Studies reporting data on gynecologic effects

Reference (year)	Transgender people concerned	Study type	Objectives	Clinical data to consider
**Loverro et al (2016)**[23]	Transmasculine individuals	Prospective observational study	To determine the type of histological and steroid receptor changes in the endometrium and myometrium of transgender men undergoing longterm testosterone therapy, prior to hysterectomy during the process of sexual reassignment surgery	Active endometrium was identified in 10 cases and secretory endometrium in two cases among transgender men receiving long-term testosterone therapy. Multifollicular ovaries were observed in cases with active endometrium.
**Hawkins et al (2021)**[24]	Transmasculine individuals	Retrospective case series	To describe clinical characteristics and associated endometrial findings of transgender and gender nonbinary people using gender-affirming testosterone	Endometrial specimens obtained after hysterectomy in individuals receiving testosterone predominantly demonstrated proliferative or atrophic patterns.
**de Blok et al (2021)**[30]	Transfeminine individuals	Prospective cohort study	To examine breast development and change in anthropometry during the first 3 years of gender-affirming hormone treatment using 3D imaging	Sustained breast growth was observed during three years of hormone therapy, and 58% of transgender women reported satisfaction with breast size.
**Borras et al (2021)**[25]	Transmasculine individuals	Prospective observational study	To evaluate the extensive endocrinological changes observed in the transgender men cohort with testosterone therapy and to evaluate the ovarian histological aspects to determine the follicular population and the stromal and cortical changes and their implications in a future fertility-preservation process	Cortical follicle distribution in transgender men receiving testosterone was within the range reported in fertile cisgender women of reproductive age.
**Anderson et al (2020)**[27]	Transmasculine individuals	Retrospective case series	To elucidate potential association of prostatic glandular proliferation within the vagina surface epithelium with androgen exposure	Thirteen cases of prostatic-type glandular proliferation were identified over 26 years, primarily involving the vaginal epithelium, in transgender men receiving long-term testosterone therapy.
**Gomez et al (2020)**[29]	Transgender and nonbinary young adults assigned female sex at birth	Qualitative study	To explore the contraceptive needs and care experiences of young trans and nonbinary people assigned female sex at birth	Participants generally relied on condoms for contraception. Knowledge gaps were reported regarding pregnancy risk during testosterone therapy and its interaction with hormonal contraceptives.
**Meyer et al (2020)**[28]	Transgender men and women	Retrospective cohort study based on medical records	To investigate efficacy and safety of hormone treatment I a large cohort of individuals under guideline-based treatment	Median time to amenorrhea was three months in transgender men receiving testosterone monotherapy. Higher hemoglobin levels at 3–4 months were associated with earlier onset of amenorrhea.

**Caanen et al (2017)**[26]	Transmasculine individuals	Single-center prospective, observational, case-control study	To assess the impact of long-term exogenous testosterone administration on the incidence of polycystic ovarian morphology (PCOM), in transgender men	The prevalence of polycystic ovarian morphology did not differ significantly between transgender men receiving testosterone and control participants.
