# Supplementary material for: Therapeutic Issues Associated with Gender-Affirming Hormone Therapy in Transgender Individuals: A Systematic Review Relevant to Community Pharmacy Practice
Source: Pharmacy (Basel). 2026 Sep 10;14(6):132. doi: 10.3390/pharmacy14060132 (PMC13610454; doi:10.3390/pharmacy14060132)
Supplement: Supplementary file 1 [file pharmacy-14-00132-s001.zip › Supplementary Table S4.pdf]

**Supplementary Table S4.** Studies reporting data on musculoskeletal effects

| Reference (year) | Transgender people concerned | Study type | Objectives | Clinical data to consider |
|---|---|---|---|---|
| **Wiik et al (2020)**[33] | Transmasculine and transfeminine individuals | Single-center observational cohort study | To explore the effects of gender-affirming treatment on muscle function, size, and composition during 12 months of therapy | After one year of gender-affirming therapy, transgender men showed substantial increases in muscle mass and strength, whereas transgender women exhibited modest changes. |
| **Boogers et al (2022)**[38] | Transgender girls | Retrospective cohort study | To investigate the effects of gonadotropin-releasing hormone analogues (GnRHa) and gender-affirming hormone therapy on growth, and the efficacy of growth-reductive treatment | Gonadotropin-releasing hormone analogues reduced growth velocity and bone maturation. Growth velocity increased following initiation of gender-affirming hormone therapy. |
| **Schagen et al (2020)**[37] | Transgender girls and boys | Prospective observational study | To describe bone mass development in adolescents with gender dysphoria treated with GnRHa, subsequently combined with gender-affirming hormones | Bone mineral density values were within the normal range at baseline. During GnRHa treatment, bone mineral apparent density stabilized or slightly decreased, and z-scores declined. Combined therapy led to significant increases in bone mineral apparent density, with normalization of z-scores in transgender boys but not in transgender girls. |
| **van Velzen et al (2020)**[40] | Transmasculine and transfeminine individuals | Prospective observational study | To study the variation of changes in body composition and characteristics associated with a lack of change | Lack of significant body composition change at 24 months occurred in 20.2% of transgender men and 9.4% of transgender women and was associated with lower testosterone levels and insufficient luteinizing hormone suppression in transgender men. |
| **Fighera et al (2018)**[35] | Transfeminine individuals | Prospective observational study | To assess bone mineral density (BMD), appendicular lean mass (ALM), and total fat mass in transwomen undergoing CSHT. | Low bone mass for age was present in 18% of transgender women at baseline. Total fat mass increased and appendicular lean mass decreased during therapy, while bone mineral density remained stable over time. |
| **Tack et al (2018)**[39] | Transgender girls and boys | Prospective observational study | To study prospectively the evolution of body composition and bone mass in late-pubertal trans adolescents using the proandrogenic or antiandrogenic progestins lynestrenol (L) and cyproterone acetate (CA), respectively. | Proandrogenic and antiandrogenic progestins induced body composition changes consistent with desired physical characteristics within one year. |
| **Pelusi et al (2014)**[31] | Transmasculine individuals | Prospective controlled study | To assess the effects of three different testosterone formulations on body weight and composition and metabolic and bone parameters. | Lean body mass increased and fat mass decreased across testosterone formulations, with no significant changes in insulin sensitivity. |

| | | | | |
|---|---|---|---|---|
| **Chrisostomo et al (2020)**[36] | Transfeminine individuals | Prospective cross-sectional study | To study bone mineral density (BMD) and body composition in transwomen undergoing cross-sex hormonal treatment (CSHT) from Brazil for over 3 years, comparing them with female and male controls | Low bone mineral density was frequently observed in transgender women and was correlated with lean body mass. |
| **Broulik et al (2018)**[34] | Transmasculine individuals | Prospective cross-sectional study | To explore the influence of long-term androgen supplementation (18 years) on bone metabolism in trans(gender) men | Hip bone mineral density in transgender men receiving long-term testosterone was higher than in cisgender females and comparable to cisgender males. |
| **Van Caenegem et al (2015)**[110] | Transmasculine individuals | Prospective controlled study | To assess the evolution of body composition and bone metabolism in transgender men during the first year of cross-sex hormonal therapy | Testosterone therapy increased muscle mass and reduced fat mass without major changes in areal or volumetric bone parameters. |
| **Van Caenegem et al (2012)**[32] | Transmasculine individuals | Cross-sectional study | To examine the effects of cross-gender sex steroid exposure on volumetric bone parameters in transsexual men | Long-term testosterone therapy was associated with higher lean body mass, greater grip strength, reduced subcutaneous fat mass, and a more masculine body fat distribution compared with female controls |
