# Supplementary material for: Therapeutic Issues Associated with Gender-Affirming Hormone Therapy in Transgender Individuals: A Systematic Review Relevant to Community Pharmacy Practice
Source: Pharmacy (Basel). 2026 Sep 10;14(6):132. doi: 10.3390/pharmacy14060132 (PMC13610454; doi:10.3390/pharmacy14060132)
Supplement: Supplementary file 1 [file pharmacy-14-00132-s001.zip › Supplementary Table S5.pdf]

Supplementary Table S5. Studies reporting data on biological effects (*: effects potentially related to drug–drug interactions)

Reference (year)	Transgender people concerned	Study type	Objectives	Clinical data to consider
**Humble et al (2019)**[111]	Transmasculine and transfeminine individuals	Retrospective cohort study	To identify the assays that should be the focus of prospective studies to determine transgender specific reference intervals	Significant changes in biochemical, endocrine, and hematologic parameters were observed at 6 and 12 months in individuals receiving masculinizing or feminizing hormones.
**SoRelle et al (2019)**[41]	Transmasculine and transfeminine individuals	Retrospective chart review	To evaluate the impact on common laboratory analytes in transgender individuals before and after initiation of hormone therapy	Red blood cell count, hemoglobin, hematocrit, and creatinine increased in transgender men and decreased in transgender women after more than six months of hormone therapy.
**Allen et al (2021)**[42]	Transmasculine and transfeminine individuals	Multicenter retrospective study	To analyze laboratory values with HT use over 5 years and after discontinuation of HT to define when values achieve stability	Red blood cell indices stabilized within six months. In transgender women, HDL and platelet levels increased beyond one year; in transgender men, LDL cholesterol and alanine aminotransferase increased and HDL decreased.
**Lim et al (2020)**[50]	Transfeminine individuals	Cross-sectional case-control study	To assess the GCA profiles of transgender women in comparison to cisgender controls and to compare how GCA differ between routes of estradiol therapy in transgender women	Transgender women exhibited hypercoagulable profiles on thromboelastography and thrombin generation assays but demonstrated increased fibrinolytic potential compared with cisgender men.
**Tack et al (2017)**[112]	Transgender girls	Retrospective study based on clinical and biochemical data	To assess the (side) effects and biochemical changes of CA alone and in combination with estrogens in adolescent transgender girls.	Hemoglobin and hematocrit decreased during cyproterone acetate therapy, and transient increases in liver enzymes were observed.
**Greene et al (2022)**[113]	Transmasculine and transfeminine individuals	Prospective cross-sectional study	To evaluate the concentration distribution of hs-cTn and NT-proBNP in a cohort of healthy transgender adults.	High-sensitivity cardiac troponin concentrations were higher in transgender men, whereas NT-proBNP concentrations were higher in transgender women.
**Caanen et al (2015)**[44]	Transgender men	Prospective observational cohort study	To investigate the effect of hormonal androgenic treatment on antimullerian hormone (AMH) serum levels in transgender men	Anti-Müllerian hormone levels decreased significantly within 16 weeks of androgen therapy.
**Herndon et al (2023)**[114]	Transgender and gender diverse individuals who received injectable estradiol	Retrospective cohort study	To compare the SC and IM estradiol doses and hormone levels in transgender and gender diverse individuals	No significant differences were observed in estradiol levels achieved with subcutaneous versus intramuscular administration.
**Defreyne et al (2020)**[115]	Transmasculine and transfeminine individuals	Multicenter prospective cohort study	To evaluate changes in measured serum estradiol levels during testosterone treatment	Serum estradiol levels decreased more prominently with long-acting testosterone undecanoate than with short-acting esters.

**Kurahashi et al (2013)**[116]	Transmasculine individuals	Prospective observational study	To conduct a dose-response analysis of testosterone replacement therapy	Serum uric acid increased after three months of testosterone therapy in a dose-dependent manner.
**Wultsch et al (2015)**[117]	Transmasculine and transfeminine individuals	Prospective observational study	To evaluate the effect of cross-sex hormone therapy in transsexuals on the capacity of serum to accept cholesterol from macrophages	The cholesterol acceptor capacity decreased in transgender women after hormone therapy and remained unchanged in transgender men.
**Cirrincione et al (2021)**[47]	Transgender women and gender nonbinary adults taking feminizing GAHT	Prospective cross-sectional study	To describe the serum estrogen profiles of three 17β-estradiol preparations, taken with or without an antiandrogen	Estrone concentrations were higher with sublingual estradiol compared with transdermal or injectable routes; injectable preparations resulted in higher estradiol concentrations than transdermal formulations.
**Kuijpers et al (2021)**[119]	Transfeminine individuals	Longitudinal observational study	To assess the lowest effective dose of cyproterone acetate in transgender women to prevent side effects.	A daily dose of 10 mg cyproterone acetate effectively suppressed testosterone levels with fewer adverse effects than higher doses.
**Spratt et al (2017)**[120]	Transmasculine individuals	Retrospective cohort study	To assess whether testosterone could be administered effectively and safely subcutaneously as an alternative to intramuscular (IM) injections.	Subcutaneous testosterone administration was effective and well tolerated across a broad age and BMI range.
**Gava et al (2020)**[46]	Transfeminine individuals	Prospective cohort study	To assess the effects of 5-year administration of cyproterone acetate (CPA) or leuprolide acetate (Leu) plus transdermal or oral estradiol (E)	Both cyproterone acetate plus estradiol and leuprolide acetate plus estradiol effectively suppressed testosterone; cyproterone acetate was associated with less favorable metabolic changes.
**Madsen et al (2021)**[43]	Transgender men	Longitudinal follow-up study	To study the prevalence and determinants in the development of erythrocytosis in trans men using testosterone.	Erythrocytosis occurred in transgender men receiving testosterone; smoking cessation, weight reduction, and switching to transdermal therapy were recommended.
**Gava et al (2016)**[45]	Transfeminine individuals	Retrospective observational study	To retrospectively compare the effectiveness and safety of 1-year administration of transdermal oestradiol (TE) with cyproterone acetate (CPA) or leuprolide acetate (Leu) in transwomen.	Cyproterone acetate and leuprolide acetate combined with transdermal estradiol were equally effective in suppressing gonadotropins and testosterone over one year.
**Gandhi et al (2020)**[121]	Transmasculine individuals	Case report	To describe a clinical case	Serum creatinine increased during testosterone therapy, likely reflecting increased muscle mass.
**Iannantuoni et al (2021)**[49]	Transmasculine individuals	Prospective observational study	To evaluate the effect of testosterone treatment on metabolic and inflammation parameters and leukocyte-endothelium interactions in transgender men	Testosterone therapy increased leukocyte–endothelium interactions and proinflammatory cytokine levels.

**Shadid et al (2020)**[48]	Transmasculine and transfeminine individuals	Multicenter prospective cohort study	To assess the long-term influences of sex hormone administration on insulin sensitivity and incretin hormones	Insulin sensitivity tended to increase with masculinization and decrease with feminization.
**Klaver et al (2018)**[122]	Transmasculine and transfeminine individuals	Multicenter prospective study	To determine the effects on body fat and lean body mass in separate body regions and on body shape after one year of hormone treatment	Hormone therapy resulted in a more feminine body fat distribution in transgender women and a more masculine distribution in transgender men.
**Tebbens et al (2022)**[123]	Transfeminine individuals	Prospective cohort study	To assess whether estrone is involved in feminization induced by hormone treatment	No association was observed between estrone concentrations and changes in body fat or breast development.
**Cunha et al (2018)**[124]	Transfeminine individuals	Retrospective register-based study	To evaluate the luteinizing hormone, follicle-stimulating hormone, testosterone, estradiol and prolactin levels after low-dose estrogen therapy with or without cyproterone acetate in transgender women	Hormone levels did not differ significantly between estrogen monotherapy and estrogen plus cyproterone acetate.
**Pappas et al (2021)**[125]	Transmasculine and transfeminine individuals	Retrospective cohort study	To determine how often testosterone and estradiol therapy alone can effectively suppress gonadal function in MTF and FTM transgender patients, and to determine the frequency and range of serum estradiol levels above the normal male range in FTM patients receiving testosterone therapy	Testosterone monotherapy achieved amenorrhea in 90.8% of transgender men. Estradiol monotherapy adequately suppressed testicular function in 84.8% of transgender women.
**Nakamura et al (2013)**[126]	Transmasculine individuals	Prospective observational study	To conduct a dose-response analysis of testosterone replacement therapy	Dose-dependent physical changes were observed after initiation of testosterone therapy.
**Wilson et al (2018)**[127]	Transmasculine individuals	Prospective, open-label, crossover study	To compare testosterone exposure and tolerability with subcutaneous versus intramuscular administration	Total testosterone exposure was comparable between subcutaneous and intramuscular administration.
**Hiransuthikul et al (2021)***[52]	Transgender women with newly diagnosed human immunodeficiency virus (HIV) infection	Prospective cohort study	To evaluate potential drug-drug interactions between hormone therapy and anti-retroviral therapy	Estradiol pharmacokinetic parameters were lower in the presence of certain antiretroviral agents, and some antiretroviral drug levels were reduced in the presence of feminizing hormone therapy.
**Van Caenegem et al (2015)**[128]	Transfeminine individuals	Prospective observational study	To examine the evolution of areal and volumetric bone density, geometry, and turnover in trans women undergoing sex steroid changes, during the first 2 years of hormonal therapy.	Bone mineral density increased and bone turnover decreased during the first two years of hormone therapy in transgender women.

**Abubucker et al (2020)*** [129]	Transmasculine individuals	Case report	Presentation of a clinical case	Enhanced bradycardia occurred with remifentanil in individuals receiving exogenous testosterone, likely due to pharmacodynamic interaction.
**Salakphet et al (2022)**[130]	Transfeminine individuals	Retrospective observational study conducted in a community-based setting	To describe the use and sex hormone concentrations of various GAHT regimens among transgender women who self-prescribe in Thailand	Self-prescribed hormone therapy did not consistently achieve target sex hormone concentrations.
**Hiransuthikul et al (2019)*** [51]	Transfeminine individuals	Prospective study	To investigate potential DDI between the hormones used in FHT and the ARV agents used for PrEP among TGW in Thailand	Plasma tenofovir exposure was reduced in the presence of feminizing hormone therapy, whereas estradiol exposure was not affected by pre-exposure prophylaxis.
**Rodriguez-Wallberg et al (2021)**[53]	Transfeminine individuals	Prospective cohort study	To assess sperm quality parameters of semen samples provided by transgender women before or after gender affirming hormone therapy (GAHT), and to compare sperm quality with a reference population of unscreened men defined by the World Health Organization	High rates of sperm abnormalities were observed, particularly among individuals previously exposed to gender-affirming hormone therapy.
