# Supplementary material for: Therapeutic Issues Associated with Gender-Affirming Hormone Therapy in Transgender Individuals: A Systematic Review Relevant to Community Pharmacy Practice
Source: Pharmacy (Basel). 2026 Sep 10;14(6):132. doi: 10.3390/pharmacy14060132 (PMC13610454; doi:10.3390/pharmacy14060132)
Supplement: Supplementary file 1 [file pharmacy-14-00132-s001.zip › Supplementary Table S6.pdf]

Supplementary Table S6. Studies reporting data on psychiatric effects

**Reference (year)**	**Transgender people concerned**	**Study type**	**Objectives**	**Clinical data to consider**
**Cantu et al (2020)**[131]	Transgender youth	Retrospective chart review	To examine changes in anxiety, depression, and suicidality from initial appointment to first follow-up in 80 transgender youth, ages 11–18	No significant change in acute distress was observed between intake and follow-up.
**Aldridge et al (2021)**[54]	Transmasculine and transfeminine individuals	Prospective longitudinal study	To investigate the effect of 18-month GAHT on depression and anxiety symptomatology and the predictors on mental health outcomes in a large population of transgender people	Depressive symptoms decreased significantly after 18 months of gender-affirming hormone therapy.
**Skewis et al (2021)**[55]	Transmasculine and transfeminine individuals	Prospective longitudinal controlled study	To assess the short-term effects (0-6 months) of the newly commencing GAHT on the quality of life and gender dysphoria experienced by trans individuals and compare these results with age-matched cisgender individuals of the same presumed sex at birth	Gender dysphoria decreased and quality-of-life measures improved within six months of therapy initiation.
**Witcomb et al (2018)**[56]	Transmasculine and transfeminine individuals	Cross-sectional study	To explore depression rates in a large sample of transgender people, compared with matched controls from the general population, as well as factors predicting depression in those taking cross-sex hormone treatment (CHT) compared to those not	Transgender individuals not receiving cross-sex hormone therapy had a markedly higher risk of probable depressive disorder compared with controls.
**Costantino et al (2013)**[63]	Transmasculine individuals	Prospective longitudinal study	To evaluate sexual function and mood of transgender men during testosterone administration after sex reassignment surgery	Sexual desire and arousal increased after one year of testosterone therapy; mood measures remained stable.
**Moffitt et al (2016)**[62]	Transfeminine individual	Case report	Description of a clinical case	Psychosis onset and relapse were temporally associated with discontinuation of non-prescription estrogen therapy.
**Allen et al (2019)**[58]	Transmasculine and transfeminine individuals	Longitudinal study	To assess the effectiveness of gender-affirming hormones for improving psychological well-being and decreasing suicidality among transgender youth	Suicidality scores decreased significantly following gender-affirming hormone therapy.
**Jones et al (2018)**[57]	Transmasculine and transfeminine individuals	Longitudinal study	To explore risk factors for eating disorder symptoms in transgender people and the role of cross-sex hormones	Eating disorder symptoms were lower among individuals receiving cross-sex hormone therapy.

**Turban et al (2020)**[59]	Transmasculine and transfeminine individuals	Cross-sectional survey study	To examine associations between access to pubertal suppression during adolescence (GnRH analogs) and adult mental health outcomes	Pubertal suppression during adolescence was inversely associated with lifetime suicidal ideation.
**Metzger et al (2019)**[60]	Transmasculine and transfeminine individuals	Controlled longitudinal study	To assess the effects of testosterone in transgender men on non-pathological personality traits over time	Neuroticism decreased and extraversion increased after testosterone therapy.
**Keo-Meier et al (2015)**[61]	Transmasculine individuals	Prospective study	To investigate the short-term effects of testosterone treatment on psychological functioning in transgender men	Testosterone therapy was associated with improvements in psychological functioning across multiple domains.
