# Supplementary material for: Therapeutic Issues Associated with Gender-Affirming Hormone Therapy in Transgender Individuals: A Systematic Review Relevant to Community Pharmacy Practice
Source: Pharmacy (Basel). 2026 Sep 10;14(6):132. doi: 10.3390/pharmacy14060132 (PMC13610454; doi:10.3390/pharmacy14060132)
Supplement: Supplementary file 1 [file pharmacy-14-00132-s001.zip › Supplementary Table S7.pdf]

Supplementary Table S7. Studies reporting data on other side effects and specific clinical situation

Reference (year)	Transgender people concerned	Study type	Objectives	Clinical data to consider
**Raj et al (2018)**[64]	Transfeminine individuals	Case report	Presentation of clinical cases	Multiple meningiomas were reported during long-term hormone therapy.
**ter Wengel et al (2016)**[65]	Transfeminine individuals	Case report with literature review	Presentation of clinical cases	Growth of multiple meningiomas was observed during prolonged estrogen exposure.
**Durcan et al (2022)**[72]	Transmasculine and transfeminine individuals	Single center, cross-sectional, web-based survey study	To investigate the relationship between GAHT and contracting COVID-19, as well as the severity of the disease in individuals with gender dysphoria	Rates of COVID-19 infection were higher in transgender men than in transgender women.
**Bourgeois et al (2016)**[5]	Transmasculine and transfeminine individuals	Nationwide retrospective pharmacovigilance study	To analyze ADRs related to gender-affirming hormone therapy reported in the French pharmacovigilance database	Few adverse drug reactions were directly attributed to hormone therapy; reported events included pancreatitis, gynecomastia, myalgia, chest pain, dyspnea, and bleeding episodes.
**Cosyns et al (2014)**[73]	Transmasculine individuals	Controlled cross-sectional study	To document voice in a large sample of transgender men, compare their vocal characteristics with those of heterosexual biological males, and determine hormonal factors with impact on their fundamental frequency.	Acoustic voice characteristics in transgender men did not differ significantly from cisgender male controls.
**Treskova et al (2018)**[66]	Transmasculine individual	Case report	Presentation of a clinical case	A case of breast cancer was reported in a transgender man receiving long-term testosterone therapy.
**Chandhoke et al (2018)**[67]	Transfeminine individual	Case report	Presentation of a clinical case	Metastatic cancer was reported in a transgender woman 15 months after initiation of estrogen therapy.
**Pang et al (2020)**[75]	Transfeminine individual	Case report	Presentation of a clinical case	Three years of GnRHa treatment initiated during adolescence was associated with reduced dysphoria and anxiety.
**Hill et al (2020)**[68]	Transfeminine individual	Case report	Presentation of a clinical case	A case of lupus nephritis was reported in a transgender woman receiving cross-sex hormone therapy.
**Garcia et al (2022)**[70]	Transmasculine individual	Case report	Presentation of a clinical case	A case of rheumatoid arthritis in remission was described in a transgender man receiving methotrexate.
**Chan et al (2013)**[69]	Transfeminine individual	Case report	Presentation of a clinical case	A case of systemic lupus erythematosus developed after long-term estrogen therapy.
**Komforti et al (2020)**[74]	Transfeminine individual	Case report	Presentation of a clinical case	Unilateral pseudoangiomatous stromal hyperplasia was observed in a transgender woman receiving estrogen therapy.

**Ocon et al (2018)**[71]	Transmasculine individual	Case report	Presentation of a clinical case	Subacute cutaneous lupus symptoms improved following testosterone therapy in a transgender man.
