# Supplementary material for: Therapeutic Issues Associated with Gender-Affirming Hormone Therapy in Transgender Individuals: A Systematic Review Relevant to Community Pharmacy Practice
Source: Pharmacy (Basel). 2026 Sep 10;14(6):132. doi: 10.3390/pharmacy14060132 (PMC13610454; doi:10.3390/pharmacy14060132)
Supplement: Supplementary file 1 [file pharmacy-14-00132-s001.zip › Supplementary Table S8.pdf]

Supplementary Table S8. Summary of treatment-related issues associated with GAHT and their potential relevance to community pharmacy practice

Clinical domain	Transfeminine individuals	Transmasculine individuals	Potential relevance to community pharmacy practice
Cardiovascular and cardiometabolic effects	VTE and stroke have been reported in observational studies; changes in lipid profiles and other cardiometabolic parameters have also been described. Rare cardiovascular events have been reported as safety signals.	Atherogenic lipid changes, obesity and changes in cardiometabolic parameters have been reported. Rare thrombotic and cardiovascular events have also been described as safety signals.	Identify cardiovascular and metabolic risk factors in the pharmacy setting, including blood pressure monitoring; review concomitant medications; provide lifestyle and risk-factor counselling, including smoking cessation counselling; and facilitate referral to appropriate healthcare professionals, including a dietitian when nutritional support is required.
Dermatologic effects	Persistent facial and body hair may occur despite feminizing therapy; hair-removal methods such as laser therapy and electrolysis are commonly used.	Acne and androgenetic alopecia may increase following masculinizing therapy.	Assess treatment-related dermatologic complaints and self-medication; provide appropriate self-care advice within scope of practice; discuss relevant treatment options and, when acne treatment is prescribed, consider potential compatibility with cosmetic hair-removal procedures and refer for medical or dermatologic assessment when required.
Gynecologic effects and sexual health	Breast development occurs progressively during feminizing therapy; fertility-related considerations remain relevant.	Amenorrhea is generally achieved after testosterone initiation; endometrial and ovarian changes, fertility considerations, pregnancy risk and contraception are relevant.	Review medications and contraceptive use; assess and reinforce treatment adherence; identify potential drug–drug interactions; provide information on pregnancy risk, contraception and fertility preservation; address sexual-health concerns sensitively; and facilitate referral when appropriate.
Musculoskeletal effects	Changes in body composition include increased fat mass and lower lean mass; bone health may require particular attention, especially in adolescents receiving GnRHa.	Increases in muscle mass, strength and lean body mass are reported, with generally preserved bone health; body composition changes may vary according to hormone exposure.	Reinforce recommended bone-health monitoring and adherence to follow-up, with particular attention to bone accrual and growth in adolescents receiving GnRHa or GAHT; discuss lifestyle measures relevant to bone and musculoskeletal health; identify situations that may warrant medical assessment.
Biological and pharmac	Changes in hematologic, metabolic and hormonal parameters occur; testosterone suppression and estradiol pharmacokinetics vary according to treatment regimen and route.	Increased hemoglobin, hematocrit and creatinine and erythrocytosis have been reported; metabolic and	Review treatment regimen, administration route and concomitant medications; identify potential drug–drug interactions and treatment-related laboratory abnormalities; reinforce appropriate

ological effects	Potential drug–drug interactions with antiretroviral therapy have been reported.	hormonal changes vary according to testosterone regimen.	monitoring and adherence; facilitate referral when medical assessment is required.
Psychiatric effects	Improvements in gender dysphoria, depression, anxiety and quality of life have been reported; rare psychiatric events have also been described.	Improvements in gender dysphoria, depression, psychological functioning and quality of life have been reported; changes in sexual desire and arousal have also been described.	Provide patient-centered and gender-affirming communication; identify mental health concerns during medication-related encounters; provide appropriate counselling and facilitate referral when psychological or medical assessment is required.
Other adverse effects and specific clinical situations	Rare events including meningiomas, malignancies, autoimmune diseases and other isolated conditions have been reported; these observations should be considered safety signals rather than evidence of causality.	Rare malignancies, autoimmune conditions and other isolated events have been reported; these observations should be considered safety signals rather than evidence of causality.	Maintain vigilance for unusual or potentially serious adverse events; review concomitant medications; provide information on warning signs and facilitate appropriate referral.

The clinical information summarized in this table is derived from the included studies. The potential relevance to community pharmacy practice represents the authors' interpretation of these findings and does not imply that the proposed pharmacist activities were directly evaluated in the included studies. Their application should be adapted to the pharmacist's national scope of practice and to the multidisciplinary care pathway.
